# Device therapy with interatrial shunt devices for heart failure with preserved ejection fraction

**DOI:** 10.1007/s10741-022-10236-8

**Published:** 2022-04-19

**Authors:** Shane Nanayakkara, David M. Kaye

**Affiliations:** Alfred Hospital and Baker Heart & Diabetes Institute, Commercial Rd, Melbourne, 3004 Australia

**Keywords:** Diastolic dysfunction, Left atrium, Shunt, Structural intervention, Atrial septal defect

## Abstract

Heart failure with preserved ejection fraction is responsible for half of all heart failure and confers substantial morbidity and mortality, and yet to date, there have been no effective pharmacologic interventions. Although the pathophysiology is complex, the primary aetiology of exercise intolerance is due to an elevated left atrial pressure, particularly with exercise. In this context, device-based therapy has become a focus. Several companies have developed techniques to percutaneously create an iatrogenic left to right shunt at the atrial level, thereby reducing left atrial pressure and reducing transmitted pressures to the pulmonary circulation and reducing pulmonary congestion. In this review, we explore the pathophysiology, evidence base, benefits, and considerations of these devices and their place in the therapeutic landscape of heart failure with preserved ejection fraction.

Heart failure with preserved ejection fraction (HFpEF) is now the most common cause of heart failure within the USA [1], in part as a consequence of the epidemiologic transition from coronary disease to aging, diabetes, and obesity. Defined as the presence of heart failure symptoms in the presence of a preserved ejection fraction (≥ 50%), HFpEF has proved itself a distinct entity from heart failure with reduced ejection fraction (HFrEF), for which a vast array of guideline-recommended therapies exist to reduce morbidity and mortality. Patients with HFpEF suffer a similar reduction in quality of life and similar mortality to those with HFrEF [2]. Despite the similar phenotype, HFpEF constitutes a heterogeneous collection of mechanisms that manifest in clinically similar ways, leading to a multitude of therapeutic failures with a variety of pharmacologic therapy. The combination of a rapid rise in prevalence combined with a lack of effective pharmacotherapy constitutes a major unmet clinical need, leading to the development of device therapy as a potential solution.

## Physiology

The dominant clinical manifestation of HFpEF is exertional dyspnoea, although fatigue, peripheral oedema, and clinical right heart failure may also co-exist. The pathophysiology of HFpEF is complex, diverse, and frequently argued [3]; the compound nature of the integration between the myocardial, microvascular, macrovascular, and comorbidity-related factors all contribute to the clinical manifestations to varying degrees. Myocardial fibrosis, a feature commonly (but not universally) seen in biopsy and autopsy studies of HFpEF [4], contributes to stiffness of both the ventricle and the atria. As a result, several clinical studies have focused on antifibrotic agents without success. The vascular endothelial pathway of nitric oxide-cyclic guanosine monophosphate/protein kinase G is also often targeted; however, none have translated to improved clinical outcomes in larger trials. Peripheral skeletal muscle wasting has been shown to impair oxygen delivery [5], with promising results from exercise therapy [6]; however, this is not feasible for significantly limited patients. Accordingly, more recent efforts have targeted the end physiologic manifestations of HFpEF, notably elevated left ventricular filling pressure, and consequently elevated left atrial pressure, particularly evident with exercise.

Pulmonary congestion, responsible for the majority of HF (heart failure) admissions, is primarily due to elevated left atrial pressure. The rapidity of the rise, and pre-existing lymphatic accommodations, leads to the variability in the relationship between left atrial pressure and the consequent development of pulmonary oedema. Prolonged elevations in left atrial pressure precede the onset of acute decompensated heart failure [7]. Elevated left atrial pressure is associated with a worse prognosis in HF overall [8], and short-term modulation improves prognosis in the short and long term [9]. In HFpEF, an elevated pulmonary capillary wedge pressure (PCWP) during exercise is associated with reduced exercise capacity, independent of cardiac output, and peripheral oxygen extraction [10].

Several studies have demonstrated the correlation of elevated left atrial pressure with exercise limitation. The 6-min walk test (6MWT) is a submaximal measure of exercise capacity frequently utilised in heart failure trials. In a study of 64 HFpEF patients [11] characterised with invasive haemodynamics, only PCWP, corrected for workload, was associated with walk distance, independent of the other haemodynamic parameters. Once indexed to workload, PCWP is strongly associated with mortality [12].

## Diagnostic considerations

To confirm the diagnosis of HFpEF, diagnostic algorithms and risk scores have been developed largely focussed on the demonstration of evidence of diastolic dysfunction and/or cardiac remodelling [13, 14], In particular, non-invasive approaches such as echocardiography or biomarker analysis (i.e., natriuretic peptides) are the most widely available tools. Whilst in many cases echocardiography can rule-in or rule-out a diagnosis of HFpEF, a considerable proportion of studies may yield indeterminate estimates of filling pressure. Left atrial strain, measured with speckle tracking, has shown promise [9, 15] in early studies although this approach is technically demanding. Under certain circumstance stress echocardiography with measurement of right ventricular systolic pressure (RVSP_ may also be of diagnostic value [16]. Echocardiography can also be limited particularly with consideration to a group of patients in whom obesity and chronic lung disease feature significantly.

Since the early 1970s, left atrial pressure has been routinely assessed via a catheter measurement of the PCWP. The current gold standard test for the diagnosis of HFpEF is exercise right heart catheterisation, whereby pulmonary pressures are measured at rest and with exercise, typically via cycle ergometry in the supine or upright position. The best surrogate for a direct left atrial pressure measurement is via measurement of the PCWP with a balloon tipped catheter; values ≥ 15 mmHg at rest or ≥ 25 mmHg with exercise are diagnostic of HFpEF [17].

## Rationale for the interatrial septal device

**Fig. 1:**
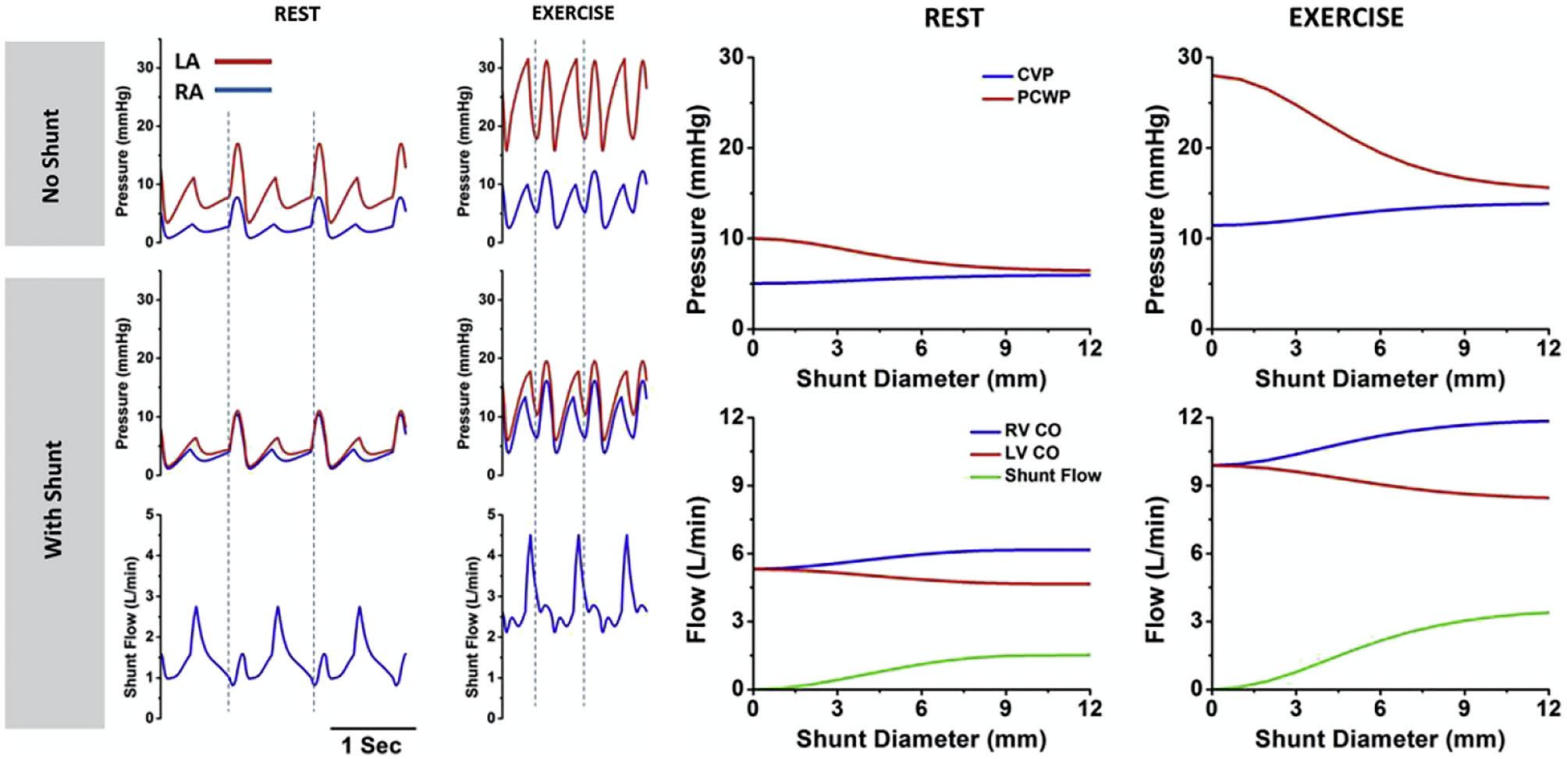
Computer modelling of the fall in atrial pressure associated with differing shunt diameters, at rest and with exercise. Figure used with permission (Kaye et al.)

Similar to HFpEF, patients with mitral stenosis also predominantly derive symptoms from an elevated left atrial pressure. In 1916, Réné Lutembacher described the combination of mitral stenosis (MS) and a congenital atrial septal defect [18], a combination later noted to attenuate and delay the progression of symptoms typically seen in patients with MS, depending on the size of the atrial septal defect. Based on this principle, the concept of an iatrogenic interatrial shunt was proposed. A small permanent opening, permitting left-to-right shunting and thereby offsetting the elevated left atrial pressures developed with exercise, would theoretically reduce the transmission of pressure to the pulmonary circulation and improve symptoms. Computer modelling studies (Fig. 1) were performed to determine the optimal shunt diameter of 8 mm [19], balancing the need for an adequate reduction in left atrial pressure against the size of the shunt itself, limited at a ratio of 1.3–1.4:1. The reduction in left atrial pressure will theoretically reduce dyspnoea and improve exercise capacity. Importantly, shunt therapies do not require ongoing compliance from the patient (to take the medication) or the clinician (to monitor pressures or weight actively), nor is there an impact of comorbid conditions or the development of resistance, as seen with diuretic therapy. Several devices have been developed (Table 1).

## Corvia interatrial septal device

**Fig. 2:**
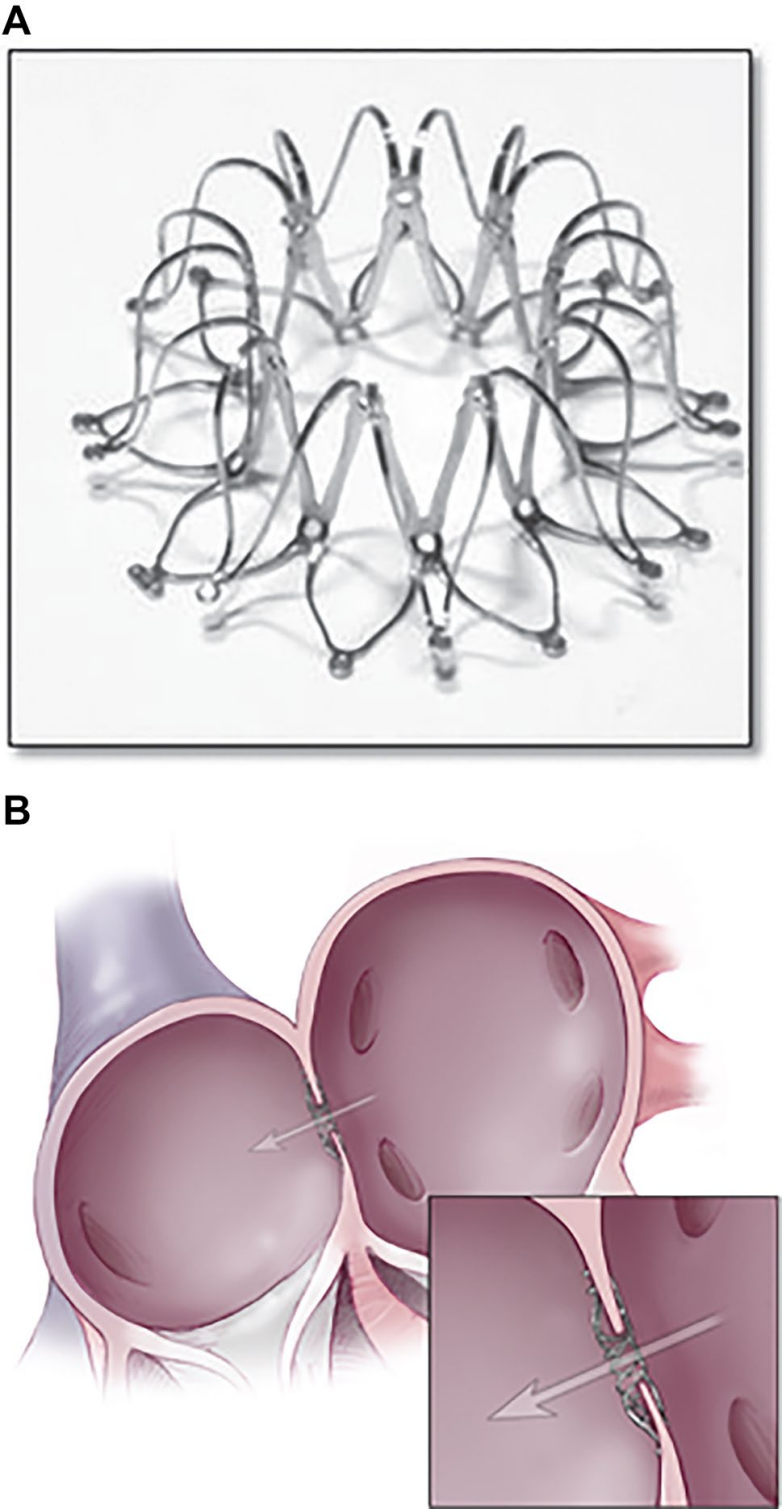
**A** The expanded Corvia IASD ex vivo. **B** The IASD in position across the interatrial septum. Figure used with permission (Sondergaard Eur J HF 2014)

These designs were then engineered to a percutaneous 8 mm nitinol interatrial shunt device (IASD, Corvia Medical, Tewksbury, MA, USA; Fig. 2), designed to be deployed to the interatrial septum from the femoral venous approach. Under conscious sedation and intracardiac echocardiography or transoesophageal guidance, transseptal puncture catheterisation is performed and a delivery system passed to the left atrium through a 16F sheath. Similar to other atrial septal devices, the left atrial aspect of the device is deployed, the system retracted to oppose the septum, and the right side deployed. The presence of left to right shunting is confirmed with echocardiography, and the patient receives a short dual antiplatelet therapy (3–6 months) followed by lifelong aspirin (Table 1).

**Table 1:** Interatrial shunt devices and key studies. RV, right ventricle; KCCQ, Kansas City Cardiomyopathy Questionnaire; PCWP, pulmonary capillary wedge pressure; 6MWD, 6-min walk distance

**Devices**	Key studies	Findings
Corvia interatrial shunt device	REDUCE-LAP HF I (NCT02600234) REDUCE-LAP HF II (NCT03088033)	Improved functional class Improved KCCQ score Reduction in PCWP
V-wave interatrial shunt device	RELIEVE-HF (NCT03499236)	Improved KCCQ score
Occlutech atrial flow regulator	PRELIEVE (NCT03030274)	Improved functional class Increased RV diameter Preserved RV function
Alleviant system	ALLEVIATE-HF-1 (NCT04583527)	Improved KCCQ Reduction in PCWP
NoYA RF-based interatrial shunt system		Increased 6MWD Improved functional class
Edwards LA-coronary sinus shunt	Alt FLOW US (NCT03523416)	Improved KCCQ score Reduction in PCWP

The IASD has been subject to several clinical trials. An initial pilot study of 11 patients demonstrated successful deployment in all patients and no major adverse cardiac events. PCWP was reduced by 28% at repeat right heart catheterisation performed at 30 days. This led to the REDUCE-LAP-HF trial, a multicenter, open-label, non-randomised study of patients with HFpEF [20]. Importantly all patients underwent clinical, echocardiographic, and invasive haemodynamic assessment as part of enrolment. Patients with significant right ventricular dysfunction were excluded. A total of 64 patients were enrolled, with no device related complications. As per the pilot study findings, the results at 6 months demonstrated improvements in functional status, quality of life, and a reduction in exercise PCWP corrected for workload. Twelve-month follow-up [21] confirmed a sustained benefit, with no echocardiographic features of worsening right ventricular function, although there was a modest increase in RV end diastolic volume.

Following on from this, a phase 2 randomised trial, REDUCE LAP-HF I [22], used a sham control against the IASD in patients with NYHA III-IV HF with an ejection fraction ≥ 40%. 44 patients were randomised, with no device related complications over 1-month follow up. Peak PCWP was reduced by 3.5 ± 6.4 mmHg in those receiving the device, compared with 0.5 ± 5.0 mmHg in the control group. The long-term follow-up out to 3 years demonstrated benefit in the device arm; however, the overall cohort was small. Across all of the existing studies, over 500 patients have received the IASD device. Device patency is 100% at 12-month follow-up. Overall survival was 82% at 4 years, with a stroke rate of 4% [23].

The pivotal multi-centre randomised controlled trial of the IASD, REDUCE LAP-HF II, has completed randomisation in late 2020 of 626 patients at 109 sites across 15 countries. The primary endpoint of the study is a composite of cardiovascular mortality, ischaemic stroke, HF admissions, or facility visits for IV diuresis, and a change in the KCCQ summary score. Analysis is expected to commence at the completion of 12-month follow-up data, with patient crossover permitted at the 24-month mark. Patients will continue to be enrolled in an open label REDUCE LAP-HF IV study at existing trial centres. Results are expected in the first half of 2022.

## The V-wave device

Another interatrial septal device has been trialled in both HFpEF and HFrEF, the V-Wave system (V-Wave, Caesarea, Israel). Several key design differences are to be noted; the device forms an hourglass shape across the septum, with a PTFE skirt over a nitinol frame, with the funnel shape designed to improve flow across a smaller central lumen (5.1 mm).

The RELIEVE-HF trial (Reducing Lung Congestion Symptoms in Advanced Heart Failure, NCT03499236) is a prospective, multicenter, randomised trial aiming to recruit 500 HFrEF and HFpEF patients, with a primary safety endpoint of major cardiovascular and neurological events, and a similar primary efficacy endpoint to the Corvia trial with a hierarchical composite of death, transplantation, LVAD implantation, recurrent HF hospitalisation, and change in KCCQ overall score. Importantly, the trial includes all patients with HF across the spectrum of ejection fraction. Patients with severe pulmonary hypertension (PASP > 70 mmHg) and right ventricular dysfunction will be excluded. The roll-in cohort have data reported [24] for 92 patients, 52% of whom had HFpEF. 99% of these patients had a successful implant, with 100% patency at 112 months. KCCQ scores increased from 45 to 56 at 12 months; in the HFpEF subset, the improvement was from 40 to 49.

## No-implant devices

One of the key issues with both above devices is the relatively large outer diameter at 19 and 14 mm respectively, potentially limiting transseptal access for future percutaneous procedures involving left atrial access. Consequently, two systems have been created specifically to generate a shunt without leaving a device. The alleviant system excises tissue using RF energy to create a 7-mm shunt. Thirty patients have been treated thus far (83% HFpEF), with observational data demonstrating improvement in KCCQ of 24 points at 6 months, and a reduction in peak exercise PCWP of 8 mmHg.

The intershunt device is a percutaneous device designed to excise a 6-mm circular section (“punch”) of tissue across the atrial septum, without leaving a permanent device in situ. This theoretically eliminates the risk of device embolisation and thrombus formation; early studies have demonstrated device patency in six patients trialled thus far [25].

The NoYA adjustable shunting system uses a radioablation catheter with a minimum size of 4 mm; however, the waist can be adjusted to be as large as 10 mm, adapted to the individual patient. The first-in-man study has enrolled 10 patients, with increases in 6-min walk distance and improvements in functional class. A larger trial of 150 patients is currently recruiting.

## Other devices

The Atrial Flow Regulator [26, 27] (Occlutech; NCT04405583) is a double nitinol disc device, available in multiple sizes based on wedge pressure and septal thickness. One-year results in both HFrEF and HFpEF patients showed improvements in functional class, with an increase in right ventricular diameter but preserved function. The Edwards shunt device [28] (Edwards Lifesciences, Irvine, California) uses a nitinol frame to create a 7-mm shunt between the coronary sinus and left atrium. In the first-in-human study, implantation was successful in 8 of 11 patients, with improvements in KCCQ and a fall in wedge of 8 mmHg. Further studies are planned.

## Risks and complications

Despite the safety profile seen thus far within the realms of the clinical trials, it is important to consider the potential for complications with these devices. All transseptal procedures carry a small but significant risk of cardiac injury, even with echocardiographic guidance. Secondly, there is a theoretical risk of right heart failure as a consequence of the shunt − early follow-up of the IASD demonstrated increased right-sided flow; however, there was no evidence of worsening RV function on follow-up out to 12 months. Atrial fibrillation, present in 30–50% of patients with HFpEF, significantly impairs exercise capacity [29]; interventions on the atrial septum could lead to increased rates of atrial fibrillation, although the reduction in left atrial pressure may also reduce the incidence. Finally, device thrombosis and paradoxical embolism is a potential consideration, although not seen in 12-month follow-up of the current devices. These concerns will require longer term follow up to determine safety. Most importantly, patient selection will be critical to determine benefit; those with a significant isolated left atrial myopathy, without other significant comorbid conditions causing exercise intolerance, will derive most benefit. In particular, patients with bi-atrial disease, where the right atrium is unable to accept the pressure shunted across, may not gain as much benefit; however, the non-invasive identification of such pathology is yet to be determined.

## Conclusions

Interatrial septal devices offer a new paradigm of treatment for patients with HFpEF by targeting the end physiologic manifestation of left atrial pressure, obviating some of the complexity around the pathophysiologic pathways leading to its development. Early pilot and randomised studies have demonstrated both safety and efficacy, with the pivotal trial of the IASD device now completed enrolment and in the follow up phase.

## Data Availability

Not applicable.
